# Sunday Funerals

**Published:** 1892-02

**Authors:** 


### SUNDAY FUNERALS.

In glancing over the pages of the *Annals of Hygiene,* we find the following absurd editorial on a very absurd law :

" SUNDAY FUNERALS IN PHILADELPHIA.—For many reasons, we can assure those who are interested in sanitary reform that they have the hearty sympathy of Archbishop Ryan, of the Archdiocese of Philadelphia, and that, whenever required, they can count upon his earnest co-operation. The latest evidence of this fact we note in the order issued against Sunday funerals, which provides that no permits will be issued for funerals to take place on Sundays, except in cases of extreme necessity.

" When, by reason of death from contagious disease, it is necessary to permit the interment of a body on Sunday, only a hearse or wagon and not more than three carriages will be allowed to enter the cemeteries."

First of all, we should like to be informed as to where the point of sanitation, or so-called sanitary reform, enters into the question of funerals taking place either Sunday, Monday or Tuesday, or upon any other day of the week.

Just here we wish to take issue with Archbishop Ryan, and denounce his proclamation as a very unjust one. It is a thrust at the workingman. For instance, here is a poor workingman, with a wife and four small children to support upon a pittance of 75 cents per day, earned, perhaps, by slaving away for sixteen or eighteen hours ; at 12 o'clock on Friday night his wife dies, and he must of necessity lose Saturday from his work. Now, by this law, he must hurry the unfortunate woman away to her last resting place almost ere he has had time to collect the thoughts running through his troubled brain, or the spirit of the dead to have wended its way from the cold and lifeless body, or else he must lose Monday, also, from his labors, and this, added to the expenses of the funeral, is unnecessarily actually taking bread from the mouths of the half-naked, half-starved children, not to speak of the possibility of his returning Tuesday only to be told that his place had to be supplied by another.

Such an abominable piece of arrogance and outraged justice should be countenanced by no intelligent and justice-loving people.

